# Heat Stress Impacts Immune Status in Cows Across the Life Cycle

**DOI:** 10.3389/fvets.2020.00116

**Published:** 2020-03-06

**Authors:** Geoffrey E. Dahl, Sha Tao, Jimena Laporta

**Affiliations:** ^1^Department of Animal Sciences, University of Florida, Gainesville, FL, United States; ^2^Department of Animal and Dairy Science, University of Georgia, Tifton, GA, United States

**Keywords:** disease, dry period, calves, heifers, lactation

## Abstract

Heat stress has a myriad of effects on dairy cattle across the life cycle. Whereas, the most commonly recognized impacts are associated with production responses, emerging evidence indicates that heat stress profoundly alters the immune response of calves and cows, from the prenatal stage through lactation. For example, *in utero* heat stress reduces passive immune transfer regardless of colostrum source, relative to normothermic conditions in late gestation. Dry cows exposed to heat stress have lower immunoglobulin responses to ovalbumin vaccination, but this effect dissipates with cooling following parturition. Conversely, cows under heat stress when dry exhibit carryover effects on the innate arm of the immune system in early lactation. In this paper we review the effects of heat stress throughout the life cycle of the dairy cow, with particular emphasis on the impact of heat stress during late gestation on the cow and the developing fetus, both before and after parturition. In addition, the impact of altered immune status under heat stress on other physiological systems, especially those supporting milk production, are considered. Finally, management interventions to prevent and reverse the effect of heat stress are presented.

## Introduction

Whereas, a number of environmental factors can affect animal immune status and productivity, heat stress dominates as a negative influence on animal health performance around the globe. As global temperatures rise, heat stress potential, both absolute, and from a duration perspective, is increasing. Heat stress occurs when a cow's ability to disperse heat produced through normal metabolism is compromised, usually as a result of ambient temperature exceeding the thermoneutral zone (TNZ), which is defined as the temperature range wherein an animal maintains normal body temperature without altering metabolic heat production or employing evaporative heat loss mechanisms. Because evaporative heat loss is affected by water vapor differences in the environment, relative humidity can also have profound effects on an animal's ability to rid the body of heat, and thus affect heat stress. Indeed, as ambient temperature and humidity increase, heat stress becomes more severe. One of the most effective indicators of the potential for heat stress, therefore, is the temperature-humidity index (THI), which accounts for the impact of rising humidity diminishing the capacity for heat loss. Of course, heat load can also increase with exposure to radiant energy (i.e., sun exposure), and so a lack of shading with a high THI can exacerbate heat stress.

Homeotherms maintain body temperature by ensuring equilibrium between heat gain and heat loss (1). Under heat stress conditions, animals acclimate to environmental heat by decreasing heat gain and increasing heat loss (1). To reduce the heat load, heat-stressed cattle decrease metabolic heat production by reducing feed intake and production, such as milk yield, and growth. Heat exchange between the animal and surrounding environment is altered in order to dissipate heat. Animals exchange heat with the environment through sensible heat transfer including radiation, convection and conduction and insensible or latent heat loss by evaporation (2, 3). The magnitude of sensible heat exchange is dependent on temperature difference between body surface of the animal and surrounding environment, and evaporative heat loss is temperature independent and associated with humidity. Radiation is of importance in animals raised in open lots and on pasture, as they receive substantial amount of heat from solar radiation during the day and dissipate heat to the cooler sky at night (1). However, for animals housed in barns or buildings, heat exchange through radiation is minimized. For example, in typical free-stall dairy barns in Florida, black globe temperature is similar to dry bulb temperature indicating that physical shade blocks most of the solar radiation (4). In response to environmental heat stress, cutaneous blood flow of cattle is increased which brings heat from the body core to the periphery and enhances heat loss through sensible heat loss (5). However, as environmental temperature increases, sensible heat exchange is minimized due to the negative temperature difference between environment and body surface and, therefore, evaporative heat loss plays the dominant role in thermoregulation of animals (6). As ambient temperature increases, evaporative heat loss is initially mediated by sweating and is then followed by increased respiratory heat loss as heat stress becomes more severe (7). As the heat load on an animal increases, various physiological systems are affected to reduce heat production and increase heat loss in an effort to maintain normal core temperature. Elevated core temperature and respiration rate are hallmarks of heat stress in cattle and other species, and are easily quantified with rectal, vaginal or ruminal probes (core temperature), and observation of inspiration and expiration via flank movements, respectively. Whereas, monitoring the impact of heat exposure is a useful management tool, there is growing evidence that the effects of heat stress can persist after the insult is removed, particularly in the developing fetus. The objective of this paper is to review the effects of heat stress on immune function and status in cows across the life cycle and provide suggestions for managing these effects to improve immune and, potentially, disease outcomes.

## Development of the Immune System in the Bovine

A robust and well-developed immune system is crucial to overcome the multitude of challenges that livestock are exposed to under the prevalent intensive production systems (8). Establishment of a functional and mature immune system in all mammals requires a sequential series of highly coordinated developmental events that begin early in embryonic/fetal life and continue through the early postnatal period (9). Development of the bovine immune system begins with conception (embryonic stage), continues progressively *in utero* (fetal stage) and reaches maturity at ~6 months after birth (10). Early studies examining bovine fetuses at different stages of development revealed that primary lymphoid organs of the immune system are present early in fetal development. The thymus is structurally present at 42 days of gestation, the spleen at 55 days, and peripheral lymph nodes are present at 60 days (11). As the fetus develops *in utero* two immune mechanisms develop: acquired (dependent on antigen recognition) and innate (independent of those recognition events). These mechanisms often act independently, however, when they act in combination, they provide greater protection than either system can alone. Ultimately, all the cells that will provide defense mechanisms originate from the same pluripotent hemopoietic stem cells.

The innate immune response mediated by phagocytic cells (neutrophils and macrophages) does not develop fully until late gestation, and in fact there is a decline in its functional capacity as gestation approaches because of the increase in fetal cortisol concentrations with the impending parturition (12). Humoral elements, such as complement proteins, are present in newborn calves; however, the levels and activity at birth are approximately reduced by half compared to those of adult cows. Also, interferon can be induced in a fetus as early as 60 days of gestation, and all the cellular components of the acquired immune response are present in fetal calves (13, 14). More specifically, the number of peripheral blood T cells dramatically decreases beginning 1 month before birth and B cells are present in much lower numbers in developing fetuses (1–2%) than mature calves (10–20%) (15, 16).

Dairy calves are immunologically naïve at birth, they are born agammaglobulinemic meaning they are relatively devoid of circulating antibodies (12). Indeed, neonatal calves have had no chance to enhance adaptive immunity by “experience” because of the protective environment *in utero*. Moreover, maternal factors at parturition, such as elevated cortisol, further depress calf immune competence. Newborn calves must obtain their antibodies from cows' colostrum via passive transfer. The immune fraction of maternal colostrum is composed primarily of antibodies but also contains cytokines, bioactive factors and cells that will activate and regulate the innate responses of calves to fight infection. Ingestion of high-quality colostrum is essential to provide neonates with immunologic protection during at least the first 2–4 weeks of life (10). It is well-recognized that a successful passive transfer of colostral immunoglobulins is crucial for calf health and survival both in the short- and long-term (17). Successful passive immunity is dependent on the content of immunoglobulin G (IgG) in the colostrum and the newborn's ability to absorb IgG (18). Colostrum-deprived calves have only trace amounts of immunoglobulins during the first 3 days of life and the risk of mortality increases significantly in the absence of passive transfer (17, 19). In fact, the levels of immunoglobulins (IgG, IgM and IgA) do not approach adult levels until 4 months after birth in colostrum-deprived calves (20). In the United States, mortality rates in pre-weaned dairy heifers are estimated to range between 8 and 11%, and more than 30% of this loss is attributed to failure of passive transfer (21).

## Fetal Programming and Heat Stress-Induced Alterations in Cell and Tissue Function

The intrauterine environment experienced by the developing fetus can shape physiological responses in preparation for postnatal life. The concept of “fetal or developmental programming” is not novel. Although initially named “the Barker hypothesis,” or “fetal and infant origins of adult disease,” after Barker's ground-breaking studies (22, 23), the concept has evolved over time after a multitude of cross-sectional, longitudinal, retrospective, and prospective studies were conducted in laboratory and livestock species. Fetal programming refers to suboptimal intrauterine conditions (i.e., nutritional, environmental, or social insults) during critical periods of fetal development that might lead to changes in tissue structure and/or function with long-term consequences on offspring physiology, metabolism, and disease susceptibility later in life [reviewed by (24)].

Fetal development during gestation is a result of highly organized, orchestrated changes primarily fueled by maternal nutrient supply to the fetus (25). Studies in humans, rodents, and livestock have confirmed the critical role of the placenta and the central role of epigenetic regulation of developmental programming (26). Prenatal malnutrition (i.e., over- or under-nutrition) experienced *in utero* is a major contributor to adverse health outcomes. Perhaps the most recognized example in livestock is the impact of intrauterine growth restriction with undernutrition on muscle development (25, 27). In dairy cattle, one well-recognized environmental factor that limits productivity and welfare is elevated ambient temperature and humidity (28, 29). Maternal exposure to high ambient temperature and humidity can create a suboptimal environment for the developing fetus. In fact, because the fetus has almost no ability to regulate its own temperature, fetal temperature is determined primarily by maternal temperature (30). It has been shown that when pregnant sheep and goats experience heat stress in late gestation, the fetus also has increased body temperature indicating a suboptimal intrauterine environment (31, 32). Historically, our group has focused on the prenatal exposure of the developing calf to hyperthermia during late gestation and the dramatic phenotypic alternations observed in postnatal performance [reviewed in (33)].

Potential underlying mechanisms explaining the effect of maternal hyperthermia on offspring development and health have been proposed. One limitation is the variability of “normal” body temperatures and lack of unique thresholds and dose-response relationships among different species, which preclude extrapolations. However, some general principles are conserved and responses can be inferred. For instance, the highly ordered progression of cellular processes that are critical to embryonic and fetal development (i.e., cell proliferation, migration, differentiation, and programmed cell death) are adversely susceptible to heat and therefore affected by elevated maternal temperatures (34). The existence of critical windows of developmental vulnerability of the embryo and fetus during prenatal phases applies to almost all mammalian species and refers to different stages when organs or tissues show different levels of heat sensitivity (35). For example, exposure of pre-implantation bovine embryos to hyperthermia *in vitro* decreases developmental competence and leads to embryonic death (36). Similar outcomes have been observed in *in vivo* studies with sheep, beef, and dairy cows (37–39), wherein early exposure to environmental heat stress results in decreased embryo viability and development and increased embryo loss. A retrospective study showed that periconceptional heat stress of dairy cows (conceived during summer months) was associated with differences in daughter milk production during their first lactation (40), indicating persistent effects of maternal stress insults early in fetal life. In most mammalian species, by the end of the embryonic stage, ~90% of the body structures are formed, followed by the fetal stage characterized by the growth of those structures. In fact, more than 75% of calf fetal growth occurs during the last 2 months of gestation (41). Thus, maternal insults (i.e., thermal stress) that occur during this stage will likely impact the maturation and growth of tissue structures and the function of cells within tissues that may lead to increased morbidity and mortality of the offspring.

Because the bovine immune system starts developing *in utero*, maternal hyperthermia during pregnancy could alter the normal developmental trajectory of immune cells and organs. Literature directly linking maternal exposure to heat stress and offspring immune outcomes is scarce in the bovine model. However, “immune system programming” has been extensively documented for a variety of prenatal maternal stressors, which can lead to changes that persist over the life course of the offspring (35, 42, 43). For example, offspring born from stressed dams have permanent alterations in their resilience to stressors and immunocompetence, primarily driven by alterations in their hypothalamic-pituitary-adrenal function (44–46). *In utero* hypothalamic-pituitary-adrenal axis programming also occurs in pigs (47). When *in utero* heat stressed pigs are exposed to postnatal stressors [i.e., handling, (48) or heat insult, (49)] they have higher cortisol concentrations and increased core temperature, respectively. It has been established that the immune system has lower priority in fetal nutrient partitioning than other systems, such as the nervous system and brain (50) which might explain the programming effect of systems regulated at the brain level (i.e., cortisol regulation and thermoregulation). Thus, hyperthermia during late gestation could be altering the nutrient partitioning to and retarding the ontogeny of the fetal immune system (i.e., the maturation and growth of lymphoid organs and immune cells). It is possible maternal hyperthermia during early stages of fetal development might impact the differentiation, migration, and/or establishment of the highly proliferative pool of lymphoid-hematopoietic progenitor cells that are destined to become immune cells; whereas hyperthermia later in development might impact growth of lymphoid organs and the cellular microenvironment impacting the development and maturation of functionally immuno-competent cells.

Another important consideration is the duration and extent of the heat insult (i.e., acute vs. chronic exposure), which might trigger distinct mechanisms to protect the fetus from hyperthermia ranging from rapid heat shock responses to suspension of protein synthesis, cell proliferation and DNA damage in both the placenta and fetus. Some of these responses seem to be conserved across mammals (34). In general, acute stressors enhance innate immunity and drive cytokine secretion, whereas chronic stress favors inflammation (43). Notably, not all fetal programming should be seen with negative connotations; in fact, resultant changes in organ structure, function, and gene expression might serve to enable the developing fetus to adapt to the environment postnatally. Understanding the programming mechanisms and implications of pre- (*in utero*) and postnatal (*ex utero*) exposure to thermal stress is critical for the development and implementation of meaningful management practices and mitigation strategies to improve productivity, health and well-being of dairy calves.

## Effects of Heat Stress During the *Prenatal* Period on Immunity

Exposure to prenatal maternal stressors during fetal development have lifelong consequences that impede the expression of the genetic potential of the offspring. Past and ongoing studies in our laboratory have revealed a series of immune alterations in the bovine calf immune response during early life and preweaning period that result from exposure to *in utero* heat stress during a relatively brief period of late gestation (i.e., the dry period of the dam).

Prenatal heat stress impairs passive transfer of colostral IgG. Newborn calves born to heat stressed dams and fed colostrum from their respective mothers have reduced apparent efficiency of IgG absorption, compared with calves born to cooled dams and receiving their dam's colostrum (51, 52). Whereas, the reduced quality of colostrum collected during summer compared with colostrum collected during other seasons has been reported, the impact of late-gestation heat stress on colostrum IgG concentration has been inconsistent in controlled studies. Some studies have reported decreased IgG (53, 54), others have observed no differences in IgG (51, 55) or even increased concentrations of IgG (56) in the colostrum of heat stressed dams. Regardless of these conflicting results, Monteiro et al. (55) demonstrated that prenatal heat stress alters the calf's capacity for IgG transfer rather than reducing the quality of the colostrum produced by the dam. Later work from our laboratory indicated that calves exposed to *in utero* heat stress have more apoptotic enterocytes in the jejunum at birth relative to calves born to cooled dams (57). In goat kids, Castro-Alonso et al. (58) provided evidence that the process of gut closure is mediated by apoptosis of enterocytes, so the acceleration of this process in heat stressed calves suggests a decrease in the capacity for postnatal Ig absorption of colostral antibodies. Heat stress exposure can compromise intestinal integrity in cows, pigs, and humans (59–61), however whether maternal heat stress causes impairment of epithelial gut function of the offspring beyond the first days of life has yet to be determined.

Prenatal heat stress reduces the overall immune competence of calves during their postnatal life by derailing hematological parameters and cellular immune status from normal. For example, calves born to heat stressed dams, receiving colostrum from their dams, had reduced circulating plasma proteins (51), red blood cell counts, platelets and circulating hemoglobin, lower lymphocyte counts, and increased circulating concentration of acute phase proteins during the pre-weaning period relative to those born to cooled dams receiving colostrum from their dams (56). Peripheral blood mononuclear cells (PBMC) were isolated from *in utero* heat stressed or cooled calves' blood during the pre-weaning stage and were incubated *in vitro* with a mitogen. A significant reduction in proliferation rate was observed in *in utero* heat stressed calves compared with *in utero* cooled calves (51, 55). Acute maternal heat stress exposure can impact the cellular immune status of the pre-weaned calf. For instance, maternal heat stress during late gestation inhibits the immune response of the offspring, by modifying T and B cell function (35). Strong et al. (62) reported a reduction in lymphocyte percentage and downregulation of blood immune markers (i.e., TNFα and toll-like receptor 2) in the first several weeks after birth of calves born to dams exposed to acute heat stress during late gestation.

Alteration of tissue morphology and function is one of the primary processes whereby developmental insults, such as hyperthermia, might exert permanent effects later in life (63). A hallmark of late gestation heat stress in the developing calf is the reduction in birth weight (51, 64–66). Intrauterine growth retardation might result from reduced uterine blood flow and nutrient availability to the placenta. Recent data from our group indicates that the placenta of heat stressed dams possess cotyledons with increased surface area, perhaps to increase the surface area for oxygen and nutrient exchange, but more likely as a mechanism to remove heat as they typically have no structural association with the fetus (67). Compromised placental vascularization is also observed in sheep, which might indicate less efficient oxygen diffusion to the growing fetus (68). A significant reduction in birth weight of *in utero* heat stressed calves was accompanied by reduction in immune organs (i.e., the thymus and spleen) relative to *in utero* cooled calves (57). Maturation of T cells, which are responsible for mounting an immune response to foreign substances, takes place in the thymus, while the spleen filters the blood and stores platelets and white blood cells. Abnormal development of these lymphoid organs can have severe consequences for an animals' adaptive immune function and ultimately compromise their health and survival. It is possible that immune outcomes are the first to be impacted under a moderate intrauterine hyperthermia and that only a more severe stress would impact other tissues and organs (i.e., liver and muscle) to influence overall birth bodyweight reductions. This hypothesis is supported by observations in severely heat stressed intrauterine growth restricted ewes that exhibit alterations in the brain and metabolic tissues resulting in a shift of fetal metabolism from anabolic to catabolic pathways (69). In addition, *in utero* heat stress also reduced the size of the liver of neonatal dairy calves (57). Even though the liver is considered a metabolic organ, it can also serve as an immune organ. In fact, *in utero* heat stress altered the hepatic methylation pattern of genes involved in immune pathways in hepatic tissue; including the major histocompatibility complex and interlukin-8 (70). The impact of intrauterine growth restriction on immunity has been reported in several other species as well. In humans, it diminishes platelets, white blood cell and neutrophil counts and the secretion of various interleukins resulting in higher risk of mortality (71). Intrauterine growth restricted piglets have decreased relative weights of the thymus, spleen, mesenteric lymph node, and reduced levels of circulating cytokines (72) and reduced total number of CD4+ and CD8+ T lymphocytes in the thymus (73).

Compromised passive transfer of immunity coupled with under-developed immune organs might impact health and reduce survival during the postnatal period. This premise is supported by recent reports from our group showing that a higher percentage of heifers that were exposed to *in utero* hyperthermia leave the herd before they reach their first lactation, compared to those that were gestated under thermoneutral intrauterine environments (74, 75). The underlying mechanisms whereby hyperthermia drives these changes *in utero* and the mechanisms resulting in compromised immune function postnatally remain to be elucidated in the bovine model. Nevertheless, epigenetic regulation, e.g., gene silencing via DNA methylation and/or histone modifications, has been proposed as one process by which environmental factors could induce long-lasting regulation of gene expression in immune cells (76).

## Effects of Heat Stress During the *Postnatal* Period on Immunity—Preweaned Calves

Although significant attention has been focused on understanding the direct impact of heat stress on lactating and more recently of dry cows [reviewed by (28) and (33)], and the development of strategies to alleviate it; pre-weaned calves are often not considered when implementing heat stress abatement. The notion that calves are less susceptible to heat stress, coupled with the lack of immediate milk production losses, might have driven the perception that cooling calves is not economically beneficial in the short-term. Consequently, study of the effects of heat stress on immune function of the neonatal calf has been less well-documented. Like mature cows, when calves exceed their ability to dissipate heat and maintain homeostasis, physiological heat stress occurs, however; the thermal neutral zone for young calves is narrower. Calves under 3 weeks of age have a thermal neutral zone between 15–25.6°C, whereas calves older than 3 weeks begin to experience heat stress at 20–21°C (77). Above these temperatures, the calf will shift energy from growth to maintain body temperature. Physiological and behavioral impairments (i.e., reduced feed intakes, increased maintenance energy needs, and lowered immunity) can lead to poor growth, higher susceptibility to disease, and in extreme cases, death. Indeed, Zhai et al. (78) found that heat stress increased autophagy in the rumen, duodenum, and kidney of bull calves at 35 days of age, suggesting that nutrient uptake capacity may be altered. Yet, temperature thresholds might vary depending on size, breed, nutrition, behavior, bedding, precipitation and humidity levels, among others [reviewed by (79)]. For instance, calves exposed to increasing temperature for 7 h, did not show signs of heat stress until temperatures approached 32°C with 60% relative humidity (80). In California, average daily temperatures above 25°C were associated with higher calf mortality (81). In addition to higher mortality rates, Heinrichs et al. (82) reported an association between calf heat stress and subsequent performance, where the higher temperatures and humidity experienced by calves resulted in higher heifer age at first calving.

The immune system of the dairy calf is compromised by direct exposure to heat stress. However, limited studies evaluate the direct impact of heat stress or heat abatement on calf immune status, and those studies vary greatly in the duration of the heat insult, the age of the animal and limited to environmentally controlled chambers. For instance, young dairy calves exposed to constant high heat (35°C) conditions showed impaired antibody- and cell-mediated immunity relative to those under thermoneutral (22.8°C) conditions (83). More specifically, after heat exposure for 3 to 14 days, circulating IgG was reduced by 27% and the number of peripheral blood lymphocytes was reduced compared with calves that were held at thermoneutrality. Acute heat stress (i.e., 37°C and 90% humidity for 12 h) exposure of post-weaned dairy calves triggers the expression of genes involved in immune responses and immunity-related signaling pathways (84). Several immune-related pathways, such as Toll-like receptor, T- and B-cell receptor signaling pathways, as well as antigen processing and presentation pathways were upregulated in the blood in response to acute heat stress. Pigs exposed to chronic heat stress (30°C) for 3 weeks during the finishing phase have reduced liver size and the hepatic protein signature indicate an induction of an innate immune response compared with thermoneutral pigs (21.7°C). These hepatic responses were observed only in heat-stressed pigs independent of reduced feed intake (85). Postnatal exposure of pigs to heat or thermoneutral conditions (32.8 vs. 23.9°C) resulted in increased neutrophil numbers and decreased antibody production (86).

## Effects of Heat Stress During the *Postnatal* Period on Immunity—Growing Heifers

The effects of heat stress on immunity in the growing heifer (i.e., post-weaning) has not been studied extensively, potentially due to a combination of the lower disease incidence of this group of animals, the notion that heifers are less impacted by heat stress compared with lactating cows, and the fact that the economic return to manage heat stress for growing heifers is not immediately evident. In a recent survey conducted in the western and eastern regions of the US (87), 5.1 and 1.0% of weaned heifers (from weaning to calving) were affected by respiratory and digestive diseases (including diarrhea), respectively, rates that are much lower relative to pre-weaned calves (12.0 and 21.1% incidences for respiratory and digestive diseases, respectively). Calving season may influence the mortality and morbidity of growing heifers. In a study conducted in small size dairy herds (<100 cows/herd) in southwest Sweden, heifers born during summer (May to August) had lower risk of mortality or improved survival from calving to 810 days of age compared with heifers calved during winter (December to April) (88). A study conducted in Ontario, Canada and Minnesota, USA, reported that heifers born during winter (December to February) had greater risk of bovine respiratory disease from 5 week to 3 months of age relative to those born during summer (June to August) (89). The impact of calving season is difficult to interpret because heifers born in one season could be at risk of getting a disease in another season, and therefore they are confounded with time.

However, the potential impact of heat stress on growing heifers should not be ignored. When exposed to elevated ambient temperature from 10 to 35°C, yearling heifers exhibit increased rectal temperature (RT), respiration rate (RR), and pulmonary ventilation rate (volume of exhaled air per min) but decreased tidal air (volume of exhaled air per respiration) (90). Those symptoms are similar to a mature heat-stressed cow and suggest that heifers also suffer from hyperthermia. The body surface area of the growing heifers increases from 1 to 12 months of age; however, the ratio of body surface area to bodyweight decreases and the heat production per unit of body surface area gradually increases as heifers grow (91, 92). These observations suggest that dissipation of metabolic heat gradually decreases and heat tolerance of the animal decreases as heifers develop. On the other hand, because heat dissipation through radiation, convection, conduction and evaporation is positively dependent on body surface area (2), the increased body surface area of growing heifers from 1 to 12 months of age also suggests increased ability to dissipate heat through sensible and latent heat loss, especially under cooling in field conditions.

Heat stress may directly impair the immunity of heifers, but reported results vary. In PBMC isolated from beef heifers of three cattle breeds, exposure to 42°C for 12 h reduces cell viability and [^3^H]-thymidine incorporation into newly synthesized DNA compared with those incubated at 38.5°C, suggesting reduced cell survival and PBMC proliferation under heat shock *in vitro* (93). Further, cells collected from Angus heifers (heat sensitive *Bos Taurus*) had greater rate of cell death when exposed to *in vitro* heat shock (42 or 45°C) relative to those collected from Brahman (heat tolerant *Bos Indicus*) and Senepol heifers (heat tolerant *Bos Taurus*), indicating a breed difference in cellular responses to heat shock *in vitro* (93). Carroll et al. (94) reported that, after an intravenous endotoxin challenge, Angus heifers (~19 months of age) exposed to heat stress conditions had greater TNFα release but lower blood INFγ concentrations compared with heifers under thermoneutrality, while heat-stressed Romosinuano (heat tolerant *Bos taurus*) heifers had lower blood TNFα concentration but greater INFγ concentration relative to thermoneutral heifers. These results further indicate a genetic impact on animal's immune responses to heat stress.

Heat stress may also disrupt the redox status of beef heifers, thereby increasing exposure to lipid hydroperoxides and other byproducts of oxidation that could harm immune cells. In Angus heifers (BW = 329 ± 14 kg) grazing tall fescue during summer, PBMC collected during hotter days in July and August had a lower ratio of reduced to oxidized glutathione, lower glutathione reductase activity but a lower glutathione peroxidase activity compared with those collected during cooler days in June, suggesting that elevated ambient temperature alters oxidative balance of immune cells (95). However, these data are confounded with the decline in forage availability and quality during the summer that also influences the oxidative redox state of immune cells (95). In beef heifers (Angus-crossbred and Charolais-crossbred, initial BW = 355 kg), providing shade in the feedlot decreases the percentage of neutrophils and increases the percentage of lymphocytes in the white blood cell population, but did not influence neutrophil chemotaxis (96). Thus, the effects of heat stress on immune function may be exacerbated by other nutritional and management interventions.

In dairy breeds, exposure of blood polymorphonuclear leukocytes isolated from Holstein heifers (310–322 days of age) growing under thermoneutral or 42°C *in vitro* reduces oxidative burst capacity as measured by cytochrome c reduction, the ability of cells to phagocytize and kill *E. coli*, and random cell migration, compared with those exposed to 38.5°C (97). Lymphocytes isolated from the same group of animals had decreased proliferative responses with or without mitogen stimulation when exposed to 42°C relative to 38.5°C *in vitro* (97). These data suggest that the function of cells of both the innate and adaptive arms of the immune system of Holstein heifers are compromised by elevated ambient temperature *in vitro*. Controlled studies that examine the impact of heat stress on immune cell function growing dairy heifers are rare. However, in a preliminary study (Bubolz, Tao and Dahl, unpublished, University of Florida), Holstein heifers (*n* = 8, 98 ± 14 days of age, BW = 128 ± 15 kg) were balanced by age and BW and randomly assigned to two environmental chambers provided with either a constant heat stress (THI = 79–82) or thermoneutral conditions (THI = 61–64). A crossover design was utilized in this experiment. Heifers were maintained on their initial treatment for 3 weeks and then switched to the opposite thermal regimen for the other 3 weeks. There was a 2 week washout period between treatment periods when thermoneutral conditions were maintained in both chambers. Peripheral blood mononuclear cells were isolated from all animals and their proliferative responses to concanavalin A were evaluated using ^3^[H]-thymidine incorporation. Neutrophil function assays including phagocytosis and oxidative burst were performed as well. During heat stress, heifers had greater (*P* ≤ 0.001) body surface temperature in the morning (35.0 vs. 30.6°C) and afternoon (36.8 vs. 31.6°C), reduced (*P* < 0.001) dry matter intake as a percentage of BW (2.29 vs. 3.83%), and increased (*P* < 0.04) water consumption (29.2 vs. 18.0 L/d) relative to thermoneutral heifers, confirming the effectiveness of the treatments. However, compared with thermoneutral heifers, heat-stressed heifers had similar (*P* > 0.60) PBMC proliferation (15136 vs. 15488 DPM, respectively), and neutrophil phagocytosis (52.7 vs. 51.2%, respectively) and oxidative burst (59.1 vs. 61.4%, respectively). These data suggest that exposure to a heat stress environment may not have a significant impact on the white blood cell functions of growing dairy heifers.

Despite the lack of conclusive evidence for heat stress to affect immune function in growing heifers, the impact may vary depending on genetic background and physiological stage of the animals, degree of heat stress, the housing facility etc. Indeed, in addition to the potential and direct impact of heat stress on heifers' immunity, one cannot ignore the importance of surround environment of animals during summer on the risk of developing a disease. For example, fly control is a critical management strategy to reduce the incidence of heifer mastitis, especially during summer (98). Dairy herds that adopt fly control programs have lower incidence of heifer mastitis compared with those without fly control (98). Heat stress may therefore act in combination to alter disease effects even in the absence of a direct input to immune function in growing dairy heifers.

## Effects of Heat Stress During the *Postnatal* Period on Immunity—Lactating Cows

Mastitis and metritis represent two of the primary pathogens induced diseases of cattle during lactation, and there is evidence that heat stress exacerbates the occurrence of both diseases. Milk somatic cell content (SCC] is an indicator of subclinical mastitis and elevations are associated with reductions in yield and processing quality (99). Using milk quality measures as a proxy for immune status, especially SCC or SC score (SCS), a number of studies support the concept that heat stress increases immune responses when compared with cooler ambient temperatures. In temperate (100, 101) and subtropical (102) environments, elevations in THI during summer months was associated with higher SCC at the herd level, and there were concomitant decreases in milk volume and quality. Whereas, overall pathogen load would be expected to increase with higher temperatures, seasonal differences exist with specific pathogens that are inconsistent with higher mastitis incidence resulting only from higher pathogen exposure. For example, Lundberg et al. (103) that in one herd monitored for 16 months, S. dysgalactiae infections were highest at the end of the summer pasture season (i.e., after heat stress) whereas *S. uberis* infections were greatest during the winter when cows were housed indoors. Therefore, it is likely that direct impacts of heat stress on host immune function plays a role in the cow's ability to resist infection. Indeed, recent study with limited heat stress abatement in early lactation cows shows improvements in immune status with cooling (104). In an arid environment with mean daily temperatures of 37°C, exposure to fans and misters from 1000 to 1800 h each day improved milk yield and dry matter intake relative to cows that received not heat abatement. Heat stress increased SCC in milk, but depressed circulating IgG and inflammatory cytokines, supporting the idea that cow immune function is adversely affected by heat stress during lactation.

A linkage of uterine disease with heat stress has been observed for decades. DuBois and Williams (105) examined seasonal incidence of retained placenta in a single herd, and found that the percentage of cows with a retained placenta (RP) doubled in the summer relative to the winter, and cows with an RP had an additional 24 days open compared with the reproductive performance of healthy cows. A recent study by Gernand et al. (106) tied the relationship of RP and metritis more closely to increases in THI. With records from 22,000 cows, puerperal disorders and RP increased linearly with the average THI in the 5 days after calving. Of interest, there was no association of the THI in the previous week on RP and metritis, suggesting that there is a period early postpartum when cows are acutely sensitive to heat stress from an immune perspective. More recent data collected from cows presenting with metritis indicates higher incidence in summer regardless of bacterial load, and greater persistency of the negative impacts of metritis on performance when it occurs in the summer vs. the winter months (107, 108). Collectively, the evidence indicates that as temperature and humidity rise, there are effects on host response to pathogens that are associated with elevated incidence of disease.

Direct evidence of heat stress effects on immune cell function in mature cows has been obtained using *in vitro* culture with direct intervention after collection of samples. Lacetera et al. (109) attempted to mimic a natural exposure of heat stress insults in PBMC collected from Holstein and Brown Swiss cows. Over 65 h in culture, PBMC were exposed to cycles through 39°C and 42°C at 13 h intervals. Expression of heat shock protein 72 (HSP72) increased with temperature, and a concomitant reduction in PBMC DNA synthesis was observed with heat stress relative to normothermia. Thus, PBMC react to temperature directly with a loss of function consistent with a reduction in immune function *in vivo*. Of interest, Holstein cows were impacted significantly less than Brown Swiss, which indicates some genetic variability in resilience to heat stress. Lecchi et al. (110) assessed the effect of *in vitro* heat stress on actual polymorphonuclear cells ability to phagocytize and generate oxidative burst activity. As heat increased in culture using a similar 39–42°C increment as previous studies, the phagocytic and burst capacity of the cells declined, evidence of a functional decline in immune system resilience with heat stress. Therefore, various immune cells suffer direct effects of heat stress on functionality, and that is likely responsible for the observed impacts on disease incidence with heat stress.

## Effects of Heat Stress During the *Postnatal* Period on Immunity—Dry Cows

The dry period, or the non-lactating interval between successive lactations, coincides with a period of elevated pathogenic and metabolic disease incidence in cattle (111). Particularly in dairy cattle, new intra-mammary infections peak around dry off and calving relative to all other phases of lactation (112). The incidence rates vary with season but are typically elevated in the summer season in comparison with winter (113). Whereas, the potential for greater pathogen loading is certainly enhanced with the warmer ambient temperatures of summer, there is substantial evidence that those same temperatures have a negative impact on the cow's immune function as well. Therefore, both exposure and response capacity are negatively impacted by heat stress.

Lacetera et al. (114) reported that moderate heat stress that increased RT and RR did not affect indicators of cell-mediated immunity. The model was a comparison of cows calving in the spring vs. summer, and thus the in this model there is confounding of heat exposure with time. Studies designed to directly compare heat stress and cooling reveal differences in immune response. Regarding the adaptive arm of the immune system, lymphocyte proliferation represents the “gold standard” *in vitro* assay of activity, and heat stressed dry cows have depressed lymphocyte proliferation relative to cooled cows (115). Heat stress decreases expression of prolactin (PRL) receptor (PRL-R) mRNA, and increases circulating PRL, suggesting that PRL signaling affects lymphocyte function directly. *In vivo*, heat stress depresses responses to antigens, which may have implications for vaccination protocols used during the dry period to prepare cows for the next lactation (116). In that study cows were immunized with chicken ovalbumin on the day of dry off, and then boosted at 14 d intervals through early lactation. Responses to each successive boost were muted in the cows during heat stress exposure in the dry period, but differences in antigen response disappeared after calving.

Evidence of direct effects of heat stress on innate immune function in the dry period is lacking, but carry-over effects, similar to those observed with lymphocyte gene expression (115), do occur. Specifically, neutrophil phagocytosis and oxidative burst activity is enhanced in cows that are cooled when dry relative to those that were under heat stress, even though all cows were cooled during lactation (116). Further, this effect alters the response to a post-partum pathogen challenge. Following treatment by cooling or heat stress in the dry period, Thompson et al. (117) observed an increase in circulating neutrophils with dry period cooling compared with heat stress. Toll-like receptor-2 (TLR-2) expression in white blood cells was greater with cooling relative to heat stress, indicating that the pro-inflammatory activity of those cells was higher given the action of TLR-2 is associated with pathogen recognition. After infusing *S. uberis* into the mammary gland, greater TLR-2 expression persisted in cooled cows, whereas heat stress increased expression of interleukin-10, which indicates more robust down regulation of the pro-inflammatory response. Thus, heat stress depresses the cow's ability to mount responses to pathogens at the cellular level.

Recent studies that examined the impact of dry period season, i.e., summer vs. winter, as a proxy for heat stress effects reveal effects on disease incidence consistent with the observed impacts on immune function. Thompson and Dahl (118) compared disease incidence with season of dry period in cows housed outdoors in Florida, and observed that dry periods corresponding to the summer months increased the incidence of mastitis, respiratory disease and displaced abomasum, suggesting that high ambient temperatures in summer reduced the immune competence of cows. Collectively, these data support the idea that heat stress in the dry period has direct and indirect suppressive impacts on immune function in the cow, which result in greater disease incidence and reduced performance.

## Management Interventions

Beede and Collier (119) proposed three approaches to alleviate the impact of heat stress: (1) modification of the environment, (2) genetic selection for heat tolerant cows, and (3) nutritional management. Because genetically altering the thermal tolerance of high producing animals is difficult (120), and there are many reviews that cover nutritional and metabolic responses and nutritional management for heat-stressed dairy cattle [i.e., (119, 121)], this section will briefly review heat abatement by physical modification of the environment. A recent analysis predicts that the milk production loss due to heat stress increases at a rate of 174 ± 7 kg/cow/decade in the 21st century, but implementation of intensive cooling using fans, water or air conditioning minimizes the loss of milk yield and is associated with significant economic return for dairy producers (122). Therefore, heat abatement is an efficient and profitable approach to alleviate the negative impacts of heat stress and is the essential component of heat stress management on dairy farms.

Heat stress abatement begins with effective determination of the heat load cows are exposed to. Temperature-humidity index is the most common measure of heat stress in the dairy industry (123). It combines the impacts of dry bulb temperature and relative humidity but does not include solar radiation and wind speed. Therefore, THI may be a good indicator of heat stress in housing structures but may not be suitable for open lot or pasture-based facilities where a black globe humidity index has been developed to incorporate the effects of solar radiation (124). Zimbleman et al. (125) reported that, when daily average THI exceeds 68, the milk yield of the high producing dairy cow starts to decrease. The THI above which the immune function of dairy cattle is influenced has not been identified. Because heat stress is defined as imbalanced thermal relation between an organism and its environment that induces hyperthermia (126), any heat abatement that reduces hyperthermia should not only improve the production performance (i.e., milk yield and growth), but also help maintain optimal immune function of dairy cattle under heat stress condition.

Providing shade effectively blocks solar radiation (127, 128), and should be utilized as the first step in heat abatement. Under moderate heat stress in a temperate climate, grazing cows exposed to shade have no differences (129) or slight decreases in body temperature during the hottest time of the day (130) relative to non-shaded cows. Consequently, cows provided with shade have similar (129) or small increases in milk yield [~3%, (130, 131)] compared with non-shaded cows. In contrast, in the subtropical climate of Florida, cooling with access to shade alone dramatically decreases body temperature and respiration rate of lactating dairy cows and improves milk yield by 11–20% (127, 128). A mature dairy cow requires 3.5 to 4.5 m^2^ of space under shade, and the shade should be 3.5–4.5 m high to minimize the radiation from shade roof to cows (123).

Besides shade, additional evaporative cooling can be implemented to further abate heat stress. Depending on the climate, evaporative cooling can be accomplished by either cooling the air or increasing the evaporative heat loss on the body surface of animals. In a dry and arid climate, evaporative cooling can be achieved through the generation of very fine water droplets that are suspended in the air and evaporate. This reduces the air temperature around cows, which improves sensible heat loss; however, the effectiveness is reduced as relative humidity increases (123). In both dry and humid climates, the combination of soakers (usually over the feedline) and fans are effective for cooling cows. Soakers or sprinklers typically deliver large water droplets through a low-pressure water line to wet the skin and hair coat of cows. The airflow provided by fans facilitates the evaporation of water from the skin, which carries heat directly away from the cows' body (132). Heat abatement using this type of active cooling system reduces the body temperature and respiration rate, increases production performance, and improves the immune function of heat-stressed cows (115, 116, 133). Alternatively, the feedline soakers may be replaced by high pressure misters or foggers placed on the face of fans. A study conducted in Kansas suggests that misters and fans reduce lactating cows' body temperature to a similar extent compared with soakers and fans (soakers operating cycle: 1 min on and 4 min off) (134). In a 3 months study conducted in southern Georgia, Weng et al. (135) reported that the daily average vaginal temperature of lactating cows cooled by misters and fans over the feedline and stalls was 0.9 °C lower (39.0 vs. 39.9°C, respectively) than those without cooling in a free stall barn.

Air movement is critical for heat abatement. In naturally ventilated barns, additional fans increase ventilation and air velocity, which enhances heat loss by convection (when air temperature is lower than cow surface temperature) and evaporation (2). Low volume, high-speed fans are effective to reduce cow body temperature when combined with soakers or misters. Brouk et al. (136) reported that air velocity provided by fans equal to or above 8 km/h at the cow level was necessary to maximize the reduction in cow body temperature when soakers are on a 1 min on and 4 min off cycle. In addition to the feedline cooling systems, fans over the freestalls are reported to further reduce respiration rate, increase intake and milk yield, and enhance lying time in stalls of lactating dairy cows (137, 138).

Compared with confinement dairies, optimal cooling strategies for grazing cattle have not been identified. As described above, providing shade is an effective approach to block solar radiation for cooling grazing dairy cattle. In some areas of the US, cows on pasture are cooled by sprinklers or misters installed on an irrigation pivot. This system is effective to reduce cow body temperature to a certain extent, but the degree of cooling is also dependent on other factors such as wind speed, relative humidity, the types of nozzles on pivots, the distance between nozzles, pressure of water, etc. Additionally, evaporative cooling using sprinklers and fans, or misters, in the holding area before milking effectively reduces cow body temperature after milking, and results in 4–5% increase in milk yield of lactating dairy cows in grazing based dairies (139–141). In some dairies, cows are often fed a partial total mixed ration in feedlots before or after milking, and appropriately designed cooling systems in the feeding area will help maintain the body temperature of cows before returning to the pasture.

Cooling dry cows should be emphasized in the heat stress management of a dairy farm because of the significant benefits on production and health of cows and calves. The more effective the cooling approach applied to dry cows, the greater the reduction in heat strain observed in cows. One early study (64) reported that providing shade to late gestation cows on pasture decreased cow body temperature (39.2 vs. 40.0°C, respectively) and respiration rate (63.3 vs. 87.4 breaths/min, respectively) compared with those without shade. A recent study (142) compared different heat abatement strategies on body temperature and respiration rate of close-up dry cows, and found that cows housed in a free stall barn cooled by feedline soakers and fans had lower average daily vaginal temperature (38.9 vs. 39.1°C, respectively) and respiration rate (43 vs. 69 breaths/min, respectively) relative to those housed on pasture with shade. Cooling during the entire dry period improves milk yield in the subsequent lactation by 4 to 5 kg/d (65, 116, 143). The length of cooling during the dry period also determines the production response of cows. Fabris et al. (66) reported that depriving dry cows of evaporative cooling (i.e., feedline soakers and fans over the free stall) during the first half, the second half, or the entire dry period equally reduced the subsequent milk yield compared with those cooled during the entire dry period. Thus, to achieve the full benefit of dry cow cooling, cows need to receive maximal cooling during both far-off and close-up periods.

Historically, the development of preventative management strategies to alleviate heat stress in calves has focused on individually housed calves (i.e., outdoor pen or hutches), where additional abatement strategies, beyond shade or different hutch materials, cannot be easily implemented. A survey conducted in the western and eastern regions of the US summarized 11 housing types for preweaned calves (87). Among the dairy operations surveyed, 37.9% used individual outside hutches and 31.8% adopted individual inside hutch/pen (87), whereas in Canada 88% of calves are housed in hutches (79). The construction materials of outside hutches influence the heat load carried by calves and that potentially affects their growth and health. For instance, plastic hutches tended to average 3–6°C higher than wooden hutches (144). Relative to hutches made of plywood, polyethylene hutches maintain greater internal air temperatures at the hottest times of a day and increase calves' respiration rate and skin and rectal temperatures in the afternoon (145). Peña et al. (146) compared polyethylene hutches with wire hutches covered by a plywood roof in the subtropical climate of Florida and reported that calves raised in polyethylene hutches had increased respiration rates and rectal temperatures in the afternoon compared with those in the wire hutches with plywood covers. Both groups of calves had similar growth during the preweaning period, but calves raised in the wire hutches showed greater incidence of respiratory diseases, as indicated by nasal discharge and coughing, and required more veterinary treatments relative to those raised in polyethylene hutches (146).

Regardless of hutch material, temperatures above 29°C inside the hutches have been reported (147). Even in moderate U.S climates, the average hutch interior can reach 22.8°C and a THI of 68 in summer, indicating severe heat stress (148). A study in Arizona reported the highest THI, highest cortisol levels, lower circulating IgG, and higher incidence of mortality in calves housed in portable-solid wall hutches, compared to improved housing systems by provision of shade and pre-cooled air (149). Two studies in the southeastern region of U.S. provided supplemental shade (80% shade cloth above the hutch) to calves housed in polyethylene hutches reporting reductions in interior temperature of the hutch, compared with hutches under direct sunlight, which translated into reductions in respiration rates (150) and better feed-to-gain ratio (151). Average elevation in respiration rates and heart rates of 20 beats/min and rectal and skin temperatures of 0.5 and 1.6°C have been observed when comparing un-shaded 7 days old bull calves with those provided with shade during a 3 days heat event (152).

Ventilation is important not only to facilitate heat abatement of calves but also minimize the occurrence of respiratory disease. In a relatively dry environment (relative humidity ranges from 15 to 70%), elevating the back of polyethylene hutches by 20 cm slightly reduces the internal ambient temperature of hutches by 0.12°C compared with the external ambient temperature and decreases the respiration rate of calves in the afternoon (153). Importantly, hutch elevation results in lower concentrations of airborne bacteria and decreases carbon dioxide levels in hutches, suggesting improved air quality (153). In a study conducted in Ohio in the summer (air temperature: max: 37°C, min: 9°C), calves were housed in wire panel hutches in a barn and cooled by fans or not from 0800 to 1700 h, to examine if forced ventilation improves calf performance (154). Compared with non-cooled calves, cooled calves had lower respiration rates and increased average daily gain and feed efficiency, suggesting that the reduced heat load improved their performance (154). But in the enclosed environment of an individual polyethylene hutch, cooling by fans may not be effective. Additionally, because convective heat loss is temperature dependent, the effectiveness of forced ventilation for cooling will be minimized when air temperature exceeds the calves' body surface temperature. Water for heat abatement (i.e., soaking or evaporative cooling) for dairy calves needs to be viewed with caution as excessive water may wet bedding material, which poses a risk for greater bacterial growth and disease development. Nevertheless, a dry and clean environment and good ventilation are basic requirements for any calf housing.

The use of automatic feeders coupled with the increased popularity of group-housing has allowed the possibility of implementing active cooling strategies to provide heat abatement more efficiently to calves. For example, increasing the area for shade (i.e., solid roof) and airflow and the option to add ventilation systems, such as natural ventilation (i.e., curtains and open walls), fans, or positive-pressure tubing are all possible with group housing but not in hutches. To date, limited research has reported benefits of cooling systems in group housed dairy calves and controlled studies describing the direct impact of heat stress exposure on immune parameters of pre-weaned group dairy calves are lacking. Emerging data from our group suggests that providing heat stress abatement to pre-weaned calves, using fans at calf-level with an average wind speed of 3.0 m/s, might positively impact their thermoregulatory ability and increase circulating levels of immune cells. Dairy calves experiencing heat stress postnatally have increased body and skin temperatures (+0.7°C and +1.7°C, respectively) coupled with depressed feed intakes which led to reduced average daily gains compared with calves with access to fans (155). Postnatal cooling improved calves' red blood cell counts and increased the percentage of circulating neutrophils relative to heat stressed calves (Laporta, unpublished data). Functional assays and quantification of specific immune cells are underway to assess whether postnatally cooling might improve immune response to vaccinations. However, even though the heat abatement applied was successful in cooling the calves postnatally, our preliminary observations indicate that it might not be enough to overcome the impact that prenatal (*in utero*) heat stress exerts on, for example, growth parameters (155).

As mentioned previously, heat abatement for growing heifers has not been studied extensively, perhaps due to the perception of low economic returns on any investment in cooling facilities. In beef heifers (Angus-crossbred and Charolais-crossbred, initial BW = 355 kg), shade structures (galvanized steel-roofed shade) reduced respiration rates of heifers, and increased feed intake and average daily gain compared with un-shaded animals during summer in Texas (96). In a study conducted in environmental chambers (air temperature = 26.1–37.8°C; THI = 76–88) using Shorthorn heifers (BW = 369 kg), continuous sprinkling on heat-stressed heifers from 1200–1600 h reduced the respiration rate from 1200–2000 h and rectal temperature from 1300–1700 h compared with those without sprinkling (156). Marcillac-Embertson et al. (157) compared the effect of two heat abatement approaches (shade vs. sprinkling) on physiology and growth of Holstein heifers housed in drylot corrals during the summer in California. The shade structures were made of solid metal sheets providing 6.5 m^2^ of shade per animal, and the sprinklers were activated 7 min every 2 h from 1,100 to 1,900 h. Compared with heifers that received sprinkling, shaded heifers had lower respiration rate, similar rectal temperature, but greater intake, average daily gain and feed efficiency. Heat abatement (i.e., shade) for growing heifers seems to be effective to reduce the thermal load of animals and improve growth performance. If extensive heat abatement such as evaporative cooling consisting of fans and sprinklers/misters would further improve the performance and future productivity (i.e., milk yield) of dairy heifers is not clear but deserves further investigation.

## Summary and Conclusions

Climate change characterized by a gradual incline in global temperature and frequent occurrence of extreme weather events is becoming a global issue that has significant impacts on performance and welfare of dairy cattle. In addition to impairment of lactational performance, heat stress has substantial impacts on a dairy cow's immune function and health at all stages of her life cycle. During lactation, the summer season is associated with increased disease incidence in cows, which may partially result from reduced immune cell function under heat stress. Recent studies provide solid evidence that dry period heat stress has significant negative effects on innate and adaptive immune function of both the dam and the offspring, influencing the morbidity and mortality of early lactation cows and calves from birth to first calving. Heat stress also has direct impacts on passive and cell-mediated immunity of neonatal calves, which again may explain increased mortality of calves during summer. Growing heifers have reductions in growth performance during hot weather, but significant impacts of hyperthermia on heifer immune function is not conclusive. Certainly, more research is required in this area.

Heat abatement by physical modification of the environment is effective and profitable to manage heat stress in dairy cattle and is the most successful way to minimize the negative impacts of heat stress on animal health. Although heat abatement for mature dairy cows, especially those housed in barns, has been extensively studied, additional effort is needed to develop effective and affordable cooling strategies for grazing dairy cattle, preweaned calves, and growing heifers to maintain immune function and optimal performance of these animals under field conditions.

## Author Contributions

GD, ST, and JL have contributed equally and made substantial, direct and intellectual contributions to the work, and approved it for publication.

### Conflict of Interest

The authors declare previously received study support from Phibro Animal Health (GD, ST, JL) and Zinpro Corp (ST). These funders had no involvement in the conception or writing of this review.
